# Ostracism Increases Automatic Aggression: The Role of Anger and Forgiveness

**DOI:** 10.3389/fpsyg.2019.02659

**Published:** 2019-12-05

**Authors:** Denghao Zhang, Sen Li, Lei Shao, Andrew H. Hales, Kipling D. Williams, Fei Teng

**Affiliations:** ^1^Department of Psychology, Renmin University of China, Beijing, China; ^2^School of Education, Renmin University of China, Beijing, China; ^3^Frank Batten School of Leadership and Public Policy, University of Virginia, Charlottesville, VA, United States; ^4^Department of Psychological Sciences, Purdue University, West Lafayette, IN, United States; ^5^Guangdong Key Laboratory of Mental Health and Cognitive Science, School of Psychology, Center for Studies of Psychological Application, The Base of Psychological Services and Counseling for “Happiness” in Guangzhou, South China Normal University, Guangzhou, China

**Keywords:** ostracism, automaticity, anger, forgiveness, implicit association test

## Abstract

While research on the “ostracism-aggression” link has focused on controlled processes in aggression, little effort has been devoted to examining the relation between ostracism and automatic aggression. Based on theories of aggression, we found that ostracized participants reported higher levels of automatic aggression than included participants (Studies 1 and 2). Furthermore, the association between ostracism and automatic aggression was mediated by anger and was especially prominent for people low in forgiveness (as compared to people high in forgiveness; Study 3). The implications of these findings are discussed.

## Introduction

Ostracism – being excluded and ignored by others – is common and pervasive. Many social animals have been observed using ostracism within their groups (Williams, 2007). On average, individuals experience one minor ostracism event daily (Nezlek et al., 2012). Although ostracism can serve a social function (Hales et al., 2016b), it is painful for those who are being ostracized (Eisenberger et al., 2003). In response to ostracism, people often engage in aggressive behaviors against those who have ostracized them and sometimes even against innocent bystanders (e.g., Twenge et al., 2001; Warburton et al., 2006; DeWall et al., 2009; DeWall and Richman, 2011; Poon and Chen, 2014).

Although the literature on the relation between ostracism and aggression is substantial, most research has focused on controlled processes in aggression. Dual-process models proposed by Shiffrin and Schneider (1977) and Gawronski et al. (2007) suggest the existence of two systems of information processing, one automatic and the other controlled. In the former, information processing is non-conscious, spontaneous, and intuitive and is mostly assessed by implicit measures; in the latter, information is processed in a conscious, controlled, and reflective manner and is assessed by self-report measures. According to dual-process models, automatic processes are as important as controlled processes for understanding human information-processing and behavior, including aggressive behaviors (Strack and Deutsch, 2003; Bluemke and Teige-Mocigemba, 2015). Moreover, implicit measures of automatic aggression are less influenced by social desirability than are self-reported measures of controlled aggression (Richetin and Richardson, 2008; Banse et al., 2015). Prior studies have consistently shown that automatic or implicitly measured aggression is a robust predictor of aggressive behaviors (e.g., Uhlmann and Swanson, 2004; Grumm et al., 2011; Banse et al., 2015; Ireland and Adams, 2015). The present research therefore aimed to empirically examine the link between ostracism and automatic aggression as well as its underlying mechanism and potential boundary conditions.

### Ostracism and Aggression

Ostracism has negative effects on a person’s affect, cognition, and mental health (Williams, 2007). An additional important negative effect of ostracism is that it increases aggression. Past research has repeatedly shown that, at least under certain circumstances, ostracism and rejection lead to overtly aggressive reactions (e.g., Twenge et al., 2001; Warburton et al., 2006; Wesselmann et al., 2010). For instance, Twenge et al. (2001, Experiments 1 and 2) found that, compared to those in a future-belonging group (who were told that they would always have friends and rewarding relationships throughout their lives), participants in a future-rejection group (who were told that they would end up alone) gave a more negative job evaluation to someone who had offended them.

Previous research on the effect of ostracism on aggression has mainly focused on factors such as cognitive disintegration, emotional numbness, and decreased self-control, which may further result in decreased prosocial behaviors and increased aggressive behavior (e.g., Twenge et al., 2001, 2003, 2007; Baumeister et al., 2005). Williams (2009) argued that ostracism can lead to aggression, especially when an individual’s senses of control and meaningful existence have been thwarted. In addition, the Multiple Motivation Model (Richman and Leary, 2009) posits that people’s reactions to ostracism are influenced by their perceptions and explanations of the ostracism. Ostracized individuals are more likely to engage in antisocial behaviors when they perceive that they are being ostracized for unfair reasons or that the broken social bond will be difficult to repair or that the bond is not important.

### Automatic Aggression and Controlled Aggression

The General Aggression Model (Anderson and Bushman, 2002) draws a distinction between impulsive behavior and thoughtful action. The former refers to an automatic and effortless route to aggression, and the latter refers to a controlled and effortful route to aggression. Compatible with this model, the Reflective-Impulsive Model (Strack and Deutsch, 2004) also contends that automatic processes (i.e., an associative system) promote the activation and execution of behavioral scripts alongside the reflective system. Research has investigated the role of automatic processes in aggression. For instance, Uhlmann and Swanson (2004) found that exposure to violent video games can lead to the automatic learning of aggressive self-concepts. Moreover, automatically activated self-concepts of aggression accounted for 11–15% variance in aggressive behavior. The reason for this might be that implicit measures of aggression (automatic aggression) are indirect measures compared to explicit measures of aggression (controlled aggression) and therefore have different relationships with objective indicators of aggressive behavior (Banse and Fischer, 2002; Banse et al., 2015).

Previous studies on the effect of ostracism on aggression have mainly focused on controlled processes in aggression, using self-report measures to assess aggressive tendencies or behaviors (e.g., Wirth, 2010, unpublished; Stenseng et al., 2014, Study 1; Wakim, 2015, unpublished; Poon et al., 2016, Study 2) and related moderating and mediating variables. For instance, aggressive response to ostracism may depend on factors such as: control deprivation (Warburton et al., 2006), rejection sensitivity (Ayduk et al., 2008), general just-world beliefs (Poon and Chen, 2014), nature exposure (Poon et al., 2016), and emotional-impulsive readiness for aggression (Rajchert et al., 2017). Further, DeWall et al. (2009) demonstrated that the link between peer rejection and aggression is mediated by a hostile cognitive bias, and Reijntjes et al. (2011) also supported this conclusion based on a study of early adolescence.

Though an important predictor of aggressive behavior, limited research has investigated the effect of ostracism on automatic aggression. The General Aggression Model (Anderson and Bushman, 2002) and Reflective-Impulsive Model (Strack and Deutsch, 2004) both assume that the mechanisms underlying the effects of social situations on aggressive behavior are often automatic in nature and that aggressive behavior is predominantly an outcome of the spontaneous appraisal of social situations (Uhlmann and Swanson, 2004; Bluemke and Teige-Mocigemba, 2015). Therefore, the present research aimed to investigate whether, and in what way, ostracism may influence automatic aggression. We hypothesized that ostracism can elicit automatic aggression through the emotion of anger. In support of our prediction, DeWall et al. (2009) found that excluded participants have higher levels of hostile cognitive bias, which is related to their aggressive treatment of other innocent people. In their studies, DeWall et al. (2009) assessed hostile cognitive bias among participants by rating pairs of words (one clearly aggressive word and one ambiguously aggressive word) for similarity (Experiment 1a) and completing word fragments with aggressive or non-aggressive words (Experiment 1b). The measures used by DeWall et al. (2009) were similar to measures of automatic aggression. However, DeWall et al. (2009) explored the mediating effect of hostile cognitive bias on the relationship between ostracism and aggression but did not examine how ostracism affects hostile cognitive bias.

### Anger as a Potential Mediator

We predicted that ostracism may increase automatic aggression, specifically, through increased feelings of anger. Previous research findings have provided partial support for this prediction. For example, Buckley et al. (2004) found that rejection triggered greater negative emotions (i.e., sadness and anger) and thus increased controlled aggression (assessed by self-report measures in Experiments 1 and 2). Chow et al. (2008) directly examined the mediating effect of anger on the link between social exclusion and antisocial behavior. In two studies, they verified the mediating role of anger in ostracism-induced aggression and suggested that the reason previous researchers did not find the mediated effect of anger is that they measured negative emotions generally, and not anger specifically. Similarly, Hales et al. (2016a) found that anger mediated the effect of ostracism on disagreeableness – a trait associated with less prosocial behavior. The above research did not focus on automatic aggression. However, Wegner and Bargh (1998) emphasized that an automatic process is not the polar opposite of a controlled process. Instead, controlled versus automatic aggression exist on a continuum, with automatic aggression effectively predicting actual aggressive behaviors. In addition, emotions (e.g., anger) are key elements that drive cognitions linked to increased levels of aggression (e.g., Ratcliff and McKoon, 1997; Bushman, 1998). For example, Matsumoto et al. (2016) also found that members of political groups who were primed with anger produced more hostile cognitions and implicit behaviors. Therefore, we predicted that ostracism, as an aversive event and source of frustration, could lead to negative emotions that trigger automatic aggression.

### Forgiveness as a Potential Moderator

Different people may respond to the same situation differently, and this applies to the experience of ostracism. According to Anderson and Bushman (2002), situational factors and personality traits can interact to determine aggressive behaviors. Previous studies have identified some factors that could moderate the effect of ostracism on aggression (Williams, 2007, 2009). For example, Williams (2009) argued that ostracism will lead to aggression, especially when an individual’s senses of control and meaningful existence have been thwarted. DeWall and Richman (2011) proposed that the potential for reconnection reduces the likelihood of ostracism-induced aggression. Poon et al. (2016) found that nature exposure can effectively weaken the link between ostracism and aggression. In the current research, we propose that forgiveness may be a crucial factor in predicting who responds to ostracism aggressively.

Forgiveness has been conceptualized not only as a specific act but also as a disposition. Individuals with high forgiveness tend to be more likely to forgive others when they are offended or hurt by transgressions (McCullough et al., 2003; Fincham et al., 2006). As a positive personality trait, forgiveness could decrease negative emotions, such as anxiety and depression (Subkoviak et al., 1995; Brown, 2003). As a psychotherapeutic intervention technique, forgiveness frees people from their anger and related negative emotions (Fitzgibbons, 1986). A large number of clinical case studies also showed that a forgiveness intervention was beneficial for individuals. It can decrease anger and lessen anxiety (Fitzgibbons, 1986; Luo and Huang, 2004). Based on this research and theory, we predicted that forgiveness would moderate the link between ostracism and anger: individuals with low forgiveness would have higher levels of anger than individuals with high forgiveness when they are ostracized by others.

## Research Overview

We hypothesized that ostracism would increase individuals’ automatic aggression and that anger would account for this effect. In Study 1, we expected that chronic ostracism would be associated with higher levels of automatic aggression. In Studies 2 and 3, we expected that experimentally induced ostracism would increase individuals’ automatic aggression. Furthermore, in Study 3, we also examined whether anger would mediate the effect of ostracism on automatic aggression and whether the mediated effect of anger would be moderated by forgiveness.

## Study 1

The goal of Study 1 was to evaluate the relationship between chronic ostracism and automatic aggression. We predicted that chronic ostracism would be positively associated with automatic aggression.

### Method

Data from 383 middle-school students (158 boys, 225 girls, mean age 16.61, SD = 1.37) in the 10th and 11th grades were used in the current study. The survey was completed in class through group administration by research staff members. All guardians of participants were notified of the survey and gave written informed consent, with signatures obtained. After the survey was completed, all participants were compensated with US $1.50 for their time.

Participants completed the Ostracism Experience Scale for Adolescents (OES-A; Gilman et al., 2013). The OES-A is an 11-item self-report instrument that measures an individual’s general perceptions of being ignored (items 2, 6, 8, 10, and 11) or excluded (items 1, 3, 4, 5, 7, and 9) by others (e.g., “In general, others treat me as if I am invisible,” “In general, others look through me as if I do not exist,” and “In general, others include me in their plans for the holidays”). The items were rated on a 5-point scale for how often (1 = *never*, 5 = *always*) participants felt the statements applied to themselves. Scores were reversed when necessary and averaged to index ostracism, with higher scores reflecting greater levels of perceived ostracism (*α* = 0.80).

Participants then completed the Chinese version of the Word Stem Completion task (Guo, 2014, unpublished) that was originally developed by Roediger et al. (1992). The Word Stem Completion task has been used to assess automatic aggression (Guo, 2014, unpublished; Nicholls, 2014, unpublished). Specifically, participants were given a list of 22 words as word stems (e.g., “刺”), and asked to fill in the missing word to form 22 phrases. Each phrase could be completed *via* an unambiguously aggressive word or a neutral word (e.g., completing “刺” with “杀” versus “绣” – “刺杀” means *assassinate*, while “刺绣” means *embroidery*). To avoid random responses, participants were given words that were not related to aggression and could not be used to form a real phrase with the stem but had a similar frequency to an unambiguous aggressive word or a neutral word (e.g., completing “刺” with “谓” – “刺谓” is not a real phrase). The frequencies of all words were determined according to the frequency dictionary of common Chinese words (Liu, 1990). Scoring was carried out by summing the number of aggression-related vocabulary items generated (out of 22). If a participant were to have chosen alternative words, indicating that they had not completed the task carefully, their data would have been omitted from subsequent analysis. However, no participants in the current study chose alternative words.

### Results and Discussion

Ostracism (*M* = 2.59, SD = 0.65) was positively correlated with automatic aggression [*M* = 9.79, SD = 4.13, *r* (381) = 0.15, *p* = 0.004, 95% confidence interval (CI) = (0.05, 0.24)]. This relationship was not reduced after controlling for age and gender [partial *r* (381) = 0.15, *p* = 0.003, 95% CI = (0.06, 0.24)]. It is worth noting that the correlation coefficients between ostracism and automatic aggression were not very high, which is unsurprising because self-reported ostracism is a controlled process, not an automatic process. In addition, the results were partly due to the variety of responses of individuals encountering ostracism (Richman and Leary, 2009) and may also have been related to the relatively young age of our participants, who would have had relatively limited experience of ostracism. The finding that ostracism is positively related to automatic aggression is consistent with our prediction that ostracism would increase the ostracized individuals’ automatic self-concept with regard to aggression but is limited by its correlational design. In Study 2, we experimentally tested whether those who are ostracized would have a higher level of automatic aggression than those who are included. In addition, in Study 2, we also adopted a different method to assess automatic aggression, namely the Aggression Implicit Association Test (Agg-IAT; Uhlmann and Swanson, 2004; Banse et al., 2015), to increase the generalizability of our findings.

## Study 2

The goal of Study 2 was to experimentally test the association between ostracism and automatic aggression by manipulating ostracism with the adapted O-Cam paradigm (Goodacre and Zadro, 2010). We expected that ostracized participants would show higher levels of automatic aggression than included participants.

### Method

#### Participants

A total of 108 Chinese undergraduate students and graduate students (44 male, 64 female; mean age = 20.71, SD = 3.56) volunteered to participate in the study. They were randomly assigned to one of two conditions (inclusion vs. ostracism).

#### Procedure

We used the O-Cam paradigm (Goodacre and Zadro, 2010) to simulate ostracism. This paradigm is fairly new and combines social and cyber ostracism. When participants entered the lab, an experimenter was pretending to test a camera according to the requirements of another experimenter on the computer. They then told the experimenter on the computer that a participant had arrived and closed the computer.

This was followed by an explanation of the procedures for the experiment, given by the experimenter. Participants were informed that they would take part in a short speech contest with two students from another university through a web conferencing program. The purpose of the research was ostensibly to explore their speech abilities on the internet. Every speaker needed to evaluate the others’ speech and give a score. Their score would subsequently influence their potential rewards.

After participants spent about 2 min preparing their speech, they began to give a short speech (1.5 min) for the other two college students through the web conferencing program. In the inclusion condition, when the participants gave their speeches, the two students on-screen attended to it and responded through timely eye contact and smiles. In the ostracism condition, at the beginning of the participant’s speech, the two students attended to it for about 15 s and then began to speak to each other and completely ignored the participant.

After finishing the speech, participants were asked to recall the process of the speech carefully and, according to their experiences, rate their level of agreement with the two statements, (“I was ignored,” “I was excluded,” *α* = 0.94) on a 5-point scale ranging from “*do not agree at all* (1)” to “*agree completely* (5)” (Williams et al., 2000). Participants with higher combined scores of the two statements had higher levels of perceived ostracism.

Finally, participants completed the Agg-IAT, which assessed response latencies while sorting stimulus words, including attribute words and target words, in a double-barreled sorting-task (Uhlmann and Swanson, 2004). The Agg-IAT is a commonly used paradigm for assessing automatic aggression. The basic rationale of the Agg-IAT is that it is easier to respond with the same response key to well-associated sorting stimulus words than less well-associated words, reflecting assumptions based on an associative social-knowledge structure (Greenwald et al., 2002). The Agg-IAT score is the difference between the average response time for the two Agg-IAT blocks (i.e., self-aggressive and others-peace versus self-peace and others-aggressive), which indicates the relative strength of automatic associations between the concept “me” and the category “aggressive.” Self-concept is broadly conceptualized as one’s perception of oneself (Shavelson et al., 1976); that is, the associations between the concept “self” and certain attributes. The Agg-IAT measures the extent to which the concept of “aggression” is associated with the concept of “self” (Richetin and Richardson, 2008).

We built a Chinese version in accordance with the previous English Agg-IAT. The concepts of “self” and “other” served as the target categories, and the attribute categories were defined as “aggressive” and “peaceful.” The Chinese version of Agg-IAT was administered on a Dell personal desktop computer (Intel Core i5–7,500 processor, Windows 8 operating system) and a 21.5-in widescreen monitor. The experiment was controlled by E-Prime 2.0 (Psychology Software Tools, Pittsburgh, PA). All stimuli were presented at the center of the screen in a 48-pt font size and printed in black characters against a white background. The category labels were presented at the top left and right corners of the screen, indicating the assigned responses (“q” or “p” keys). The labels were “self” and “other” for the target categories and “aggressive” and “peaceful” for the attribute categories. Participants sat about 50 centimeters away from the screen. At the beginning of the Agg-IAT, participants were asked to respond as quickly as possible and to make as few mistakes as possible.

The Agg-IAT in this experiment consisted of seven blocks. In the first block (20 trials), participants practiced categorizing “self” stimuli (using the “q” key) and “other” stimuli (using the “p” key). In the second block (20 trials), participants practiced discriminating between “aggressive” (the “q” key) and “peaceful” stimuli (the “p” key). In the third block (20 trials), the categories “self” and “aggressive” (the “q” key) as well as “other” and “peaceful” (the “p” key) shared the same response keys; Block 4 (40 trials) was the same as Block 3, but it served as the first critical dependent-variable block; in Block 5 (20 trials), participants again practiced making a discrimination between “aggressive” (the “p” key) and “peaceful” stimuli (the “q” key) with the reverse keys from Block 2. Finally, in Blocks 6 and 7, the response keys were assigned as follows: “self” and “peaceful” to the “q” key and “other” and “aggressive” to the “p” key. The last block served as the second critical dependent-variable block. All participants went through the seven blocks in an identical sequence, but in each block, the presentation order of stimuli was randomized. Each trial started with a fixation cross at the center of the screen for 750 milliseconds, followed by the imperative stimulus with the category labels at the top left and right corners. The stimulus remained on the screen for 3,000 milliseconds or until a response was made. If the response was incorrect, the error message “Error!” was presented for 500 milliseconds; if there was no response, the message “Faster” was presented, and if the response was correct, a blank screen appeared for 500 milliseconds. Reaction Time was the interval between the onset of the stimulus and the depression of a response key. Response accuracy was also recorded for each trial.

Following Greenwald et al. (1998), we deleted trials with latencies longer than 3,000 ms or shorter than 300 ms and replaced error responses with the mean latencies plus 600 ms. The D scores were calculated by subtracting the mean latency in Block 4 from the mean latency in Block 7. Higher D scores indicated a stronger association between the self and aggression, which represented higher automatic aggression. Participants were then fully debriefed and received US$1.50 as compensation.

### Results and Discussion

#### Manipulation Checks

The manipulation was successful. Ostracized participants felt significantly more ostracized (*M* = 3.26, SD = 1.23) than included participants (*M* = 1.79, SD = 1.05), with *t* (104) = 4.47, *p* < 0.001, and *d* = 1.29.

#### Automatic Aggression

Using the experimental condition (inclusion vs. ostracism) as the independent variable and the D scores on the Agg-IAT as the dependent variable, we conducted an independent-sample *t-*test analysis to determine whether automatic aggression varied across experimental conditions. The automatic aggression level in the ostracism condition (*M* = 10.76, SD = 105.84) was higher than the level in the inclusion condition (*M* = −34.85, SD = 124.36), with *t* (106) = 2.27, *p* = 0.025, and *d* = 0.21.

Therefore, as predicted, ostracism increased the ostracized individuals’ automatic aggression. In Study 3, we attempted to replicate the results of Study 2 with a different ostracism paradigm known as the Get-Acquainted Paradigm (Nezlek et al., 1997). Furthermore, Study 3 also focused on the process through which ostracism would increase individuals’ automatic aggression. Based on the General Aggression Model (Anderson and Bushman, 2002), the Cognitive Neoassociation Theory (Berkowitz, 1990), and related research concerning the relationship between ostracism and aggression (e.g., Chow et al., 2008; Hales et al., 2016a), Study 3 tested a potential mediating effect of anger on the link between ostracism and automatic aggression. In addition, people with high forgiveness have lower levels of anger; thus, Study 3 also tested the potential moderating effect of forgiveness in this process.

## Study 3

Study 3 aimed to replicate and extend the findings of Study 2 by testing the underlying mechanism of the effect of ostracism on automatic aggression. Specifically, we predicted that anger would mediate the relationship between ostracism and automatic aggression and that ostracized people who are high in forgiveness would report less anger than those who are low in forgiveness.

In Study 3, participants first completed a measure of trait forgiveness (Berry et al., 2005; Zhang and Luo, 2011). Next, participants were divided into ostracized and included groups by the Get-Acquainted Paradigm (Nezlek et al., 1997). Finally, participants completed a measure of state anger and the same Agg-IAT as in Study 2.

### Method

#### Participants

A total of 112 college students (47 male, 65 female) from a university located in Beijing, China, participated in this experiment. The mean age of participants was 21.34, with an SD of 4.03. All participants were compensated with US $1.50 for their time.

#### Procedure

At the beginning of the experiment, the participants completed the Trait Forgiveness Scale (Berry et al., 2005; Zhang and Luo, 2011). This scale is a 10-item self-report measure designed to assess an individual’s disposition to forgive interpersonal transgressions. Participants responded to 10 items on a 5-point rating scale (1 = strongly disagree, 5 = strongly agree). The respondents indicated their agreement with each of the items (e.g., “People close to me probably think I hold a grudge too long,” “I can forgive a friend almost anything”). The 10 scores were averaged into an index of forgiveness (*α* = 0.77).

Next, we used the Get-Acquainted Paradigm (Nezlek et al., 1997) to activate ostracism. All participants were divided into 20 groups, each group consisting of 4–8 participants of the same gender who were not acquainted with each other before arriving at the lab. Participants were informed that they would complete some tasks together with their partners, so they could talk for 15 min to get to know more about each other. After 15 min, the participants were led to separate rooms, and each of them was asked to choose a partner from among the other participants with whom they had taken part in the immediately preceding discussion and then to write down the partner’s name. The experimenter collected these lists and returned to the lab several minutes later to report the results. Participants were randomly assigned to an ostracism condition or a social inclusion condition. The participants who were included were told, “Congratulations! Everyone chose you as the partner they’d like to work with.” Those who were ostracized were told, “I hate to tell you this, but no one chose you as the partner they’d like to work with” (Twenge et al., 2001). Following this, all participants were told, “You have to complete some surveys regarding your own current feelings before taking part in a formal activity.”

Participants then rated their feelings on a 5-point scale, ranging from “do not agree at all (1),” to “agree completely (5),” on the same two questions as in Study 2 (“I was ignored,” “I was excluded,” *α* = 0.95).

Afterward, state anger was measured with the State–Trait Anger Inventory-2 (Spielberger, 1999); participants indicated how they felt “right now” on 12 statements (e.g., “I feel annoyed,” “I feel angry”) on a 5-point scale (1 *= not at all*; 5 = *very much so*; *α* = 0.83).

Finally, participants completed the same Agg-IAT as in Study 2 and were then debriefed and thanked.

### Results and Discussion

#### Manipulation Checks

The manipulation of ostracism was effective. The ostracized participants felt significantly more ostracized (*M* = 4.24, SD = 2.51) than included participants (*M* = 2.35, SD = 1.48), *t* (110) = 3.06, *p* = 0.003, *d* = 0.92.

#### Bivariate Analysis

Means, standard deviations, and correlations for anger, forgiveness, and automatic aggression are presented in Table 1.

**Table 1:** Means and standard deviations of the variables in Study 3.

Measures	*M* (SD)		Anger	Forgiveness
	Included (55)	Ostracized (57)		
Anger	1.12 (0.19)	1.29 (0.30)	1	
Forgiveness	2.69 (0.52)	2.56 (0.33)	−0.33^*^	1
Automatic Aggression	−71.66 (112.92)	8.43 (104.43)	0.37^**^	−0.28^**^

* p < 0.05;

** *p < 0.01*.

#### Anger as a Mediator of Ostracism and Automatic Aggression

**Figure 1:**
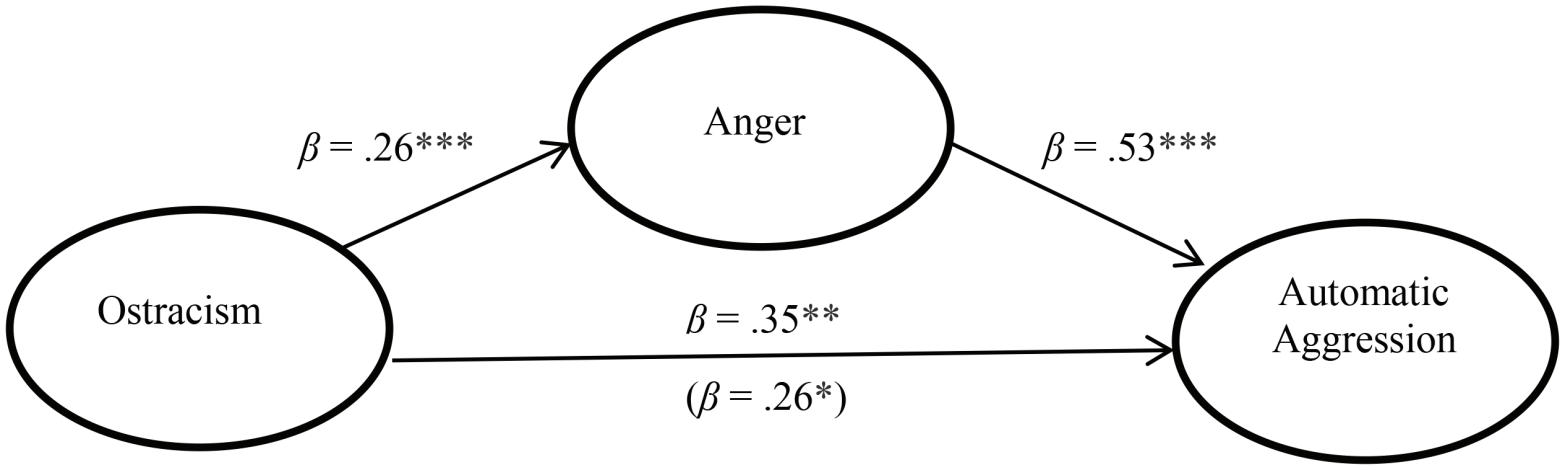
Anger partially mediates the effect of ostracism on automatic aggression in Study 3. Note: *p < 0.05; **p < 0.01; ***p < 0.001.

To examine the role of state anger in the relationship between ostracism and automatic aggression, we performed a mediation analysis using the bootstrap procedure (Hayes, 2013). The experimental condition was coded as 1 (ostracism) or − 1 (social inclusion). Figure 1 shows the mediation model. The effect of ostracism remained significant (from *β* = 0.35, *p* = 0.011 to *β* = 0.26, *p* = 0.031) when anger was included in the equation. The 95% bias-corrected confidence interval for the indirect effect did not include zero ([0.07, 0.35]), suggesting a significant indirect effect, which was 0.14. Because the effect was not reduced to non-significance, it appears that anger partially mediates the effect of ostracism.

#### Moderated Effect of Forgiveness on the Mediated Effect of Anger

In order to test the moderating effect of forgiveness in this model of ostracism, anger, and automatic aggression, we performed a test of moderated mediation using the PROCESS Macro (Hayes, 2013, 2015). The results are presented in Table 2. Forgiveness moderated the relation between ostracism and anger (*β* = −0.94, *t* (108) = −5.55, *p* < 0.001) but did not moderate the relation between ostracism and automatic aggression or between state anger and automatic aggression.

**Table 2:** The moderating effect of forgiveness in Study 3.

Dependent	Independent	*R*^2^	*F*	*β*	CI	*t*
Anger	Ostracism	0.36	20.68^***^	0.51	(0.20, 0.82)	3.27^***^
	Forgiveness			0.0001	(−0.18, 0.18)	0.00
	Ostracism × Forgiveness			−0.94	(−1.27, −0.60)	−5.55^***^
Automatic Aggression	Ostracism	0.23	6.41^***^	0.54	(0.18, 0.91)	2.96^**^
	Anger			0.25	(0.04, 0.49)	2.10^*^
	Forgiveness			−0.06	(−0.29, 0.17)	−0.51
	Ostracism × Forgiveness			−0.26	(−0.72, 0.19)	−1.16
	Forgiveness × Anger			−0.12	(−0.11, 0.36)	1.04

* p < 0.05;

** p < 0.01;

*** *p < 0.001*.

**Figure 2:**
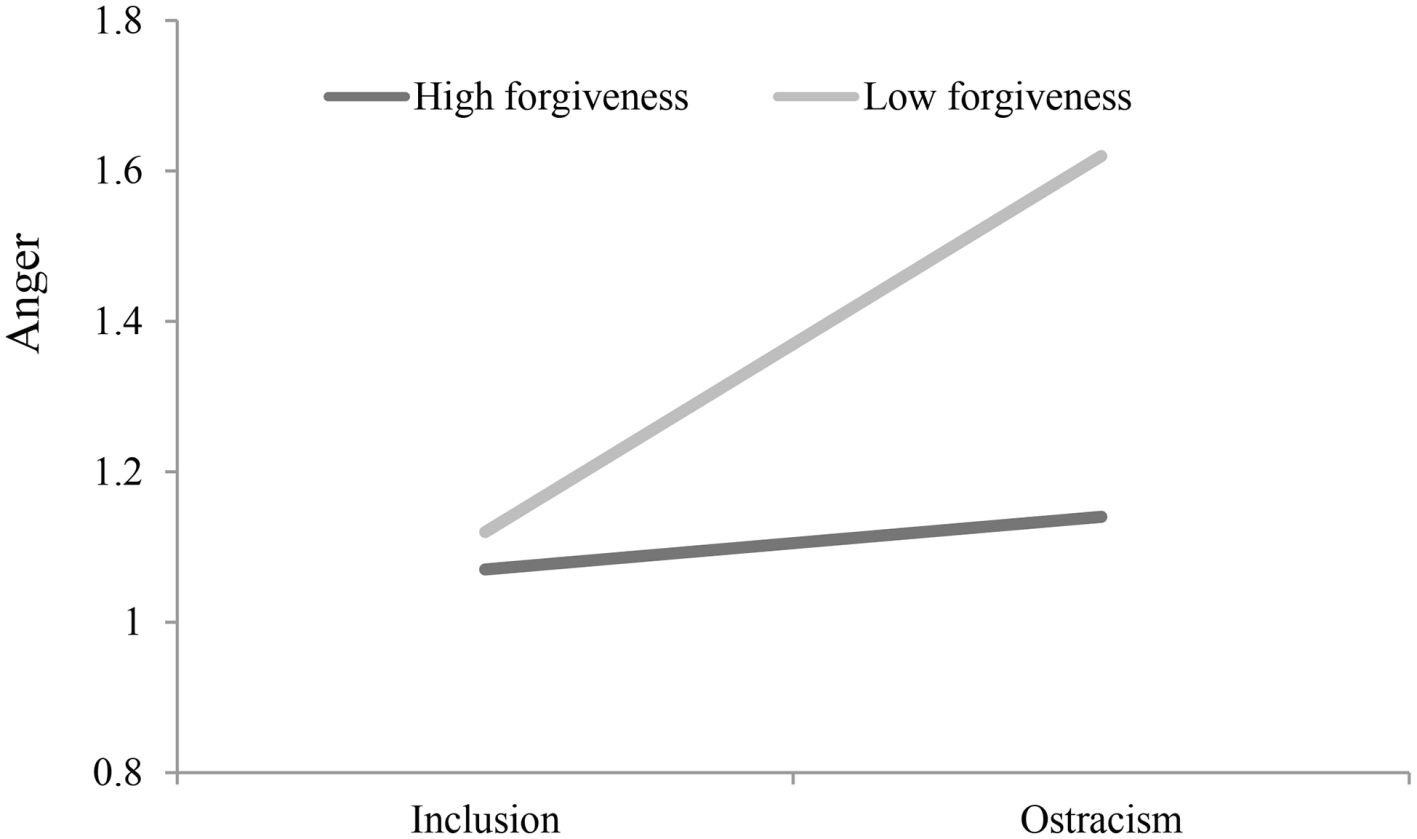
Effects of ostracism and forgiveness on anger in Study 3.

We used the simple slope for the regression of anger on ostracism by using the low (one standard deviation below the mean) and high (one standard deviation above the mean) values for forgiveness. As Figure 2 shows, there was a significant positive effect of ostracism on anger at low levels of forgiveness (*ß*_simple_ = 0.62, *p* = 0.012). The effect of ostracism on state anger at high levels of forgiveness was positive but not significant (*ß*_simple_ = 0.37, *p* = 0.552).

Study 3 indicated that the indirect relation between ostracism and automatic aggression *via* anger was moderated by forgiveness. Specifically, the relation between ostracism and anger became non-significant for high-forgiveness individuals.

## General Discussion

Based on the findings of the three studies, we concluded that ostracism increases the ostracized individuals’ automatic aggression and that this link is mediated by anger. Moreover, a crucial boundary condition of this mediated effect was observed such that ostracism only increased the anger of people with low forgiveness but not those with high forgiveness.

Our findings advance our understanding of ostracism by investigating its impact on automatic aggression. It is useful to distinguish between two different types of aggression in terms of the level of control versus automaticity (Richetin and Richardson, 2008; Ireland and Adams, 2015). Past research has mainly focused on the harmful effects of ostracism on the controlled process in aggression. Our research demonstrated that ostracism also triggers higher levels of automatic aggression. By what mechanism does ostracism lead to automatic aggression? According to Uhlmann and Swanson (2004), in Studies 2 and 3, two priming ostracism episodes may have temporarily increased the automatic accessibility of an aggressive-possible self. If one suffers from long-term ostracism, this temporary aggressive self may become an actual self, which is partially supported by Hales et al. (2016a) and Study 1, although Study 1 is not causal but correlational research. As a part of the self-concept, such automatic associations between self and aggression could guide how we perceive situational stimuli and respond to them (Anderson and Bushman, 2002; Uhlmann and Swanson, 2004). Furthermore, Study 3 found that anger played a mediating role in the relationship between ostracism and automatic aggression. As Bluemke and Teige-Mocigemba (2015) suggested previously, the influence of situational factors on aggressive behavior is achieved by activating emotions such as anger and hostility, and the effect of the emotions on behavior is achieved by activating specific knowledge structures and behavioral scripts. Therefore, the present investigation contributes to the related research on anger.

Furthermore, we used the Word Stem Completion Task (Study 1) and Agg-IAT (Studies 2 and 3) to measure automatic aggression. The two methods are useful and valid implicit measures of one’s automatic self-concept with regard to aggression (Anderson et al., 2003; Uhlmann and Swanson, 2004; Banse et al., 2015; Lemmer et al., 2015). The idea behind the two indirect methods to assess automatic aggression is characterized by assumptions regarding an associative social-knowledge structure. This structure helps us to process information about ourselves and the world (Greenwald et al., 1998). Although they do not measure actual aggressive behavior, this cognitive association between self and aggressive traits does predict actual aggressive behavior and has incremental validity over explicit measures (Richetin and Richardson, 2008). Intention to hurt targets is a topic of disagreement among scholars who study aggression; given that automatic aggression could drive aggressive behavior, the intention of hurting may also be activated unconsciously, and the individual may not be aware of it (Richetin and Richardson, 2008). Furthermore, aggressive behaviors inside the laboratory do not necessarily generalize to situations outside the laboratory (Warburton et al., 2006), and controlled aggression is limited by social desirability concerns (Banse et al., 2015). As a result, to enhance our understanding of ostracism, we must better understand automatic aggression as an outcome of ostracism. Of course, there remain unanswered questions regarding implicit measures of aggression, leaving room for improvement. In particular, the prediction of aggressive behavior by implicit measurement is also affected by individual differences and the situation in which the aggressive behavior occurs (Richetin and Richardson, 2008).

Our research in this regard also identifies a boundary condition with respect to the effect of anger. Indeed, our research suggests that ostracism produces increased anger depending on the ostracized individual’s level of forgiveness. When individuals are ostracized, those with high forgiveness experience relatively lower anger, whereas those with low forgiveness experience high anger. According to Worthington’s model of forgiveness (Worthington and Wade, 1999), forgiveness can facilitate the reduction or replacement of immediate angry emotions with positive emotions (McCullough et al., 2003; Berry et al., 2005) and is an emotion-focused coping strategy (Worthington and Scherer, 2004) that serves as a buffer against perceived stress (Friedberg et al., 2005). This could explain why forgiveness moderates the link between ostracism and anger but not the link between ostracism and automatic aggression. The results can be adequately explained by the General Aggression Model (Anderson and Bushman, 2002): anger is created by a situational factor (ostracism) and a dispositional factor (forgiveness), which in turn determines the final behavior. This result is in line with previous studies that suggest that forgiveness interventions can reduce anger efficiently and eventually decrease aggression (Hebl and Enright, 1993; Al-Mubak et al., 1995).

Our research is not without limitations, which we believe can be further addressed in future research. One limitation in the present research is that, although automatic aggression could reveal association of self and intention to harm (Richetin and Richardson, 2008), the incremental validity of automatic aggression could not be evaluated because we did not include measures of explicit aggression in our studies. We believe that it would be worthwhile to examine whether automatic aggression can account for effects independent of explicit measures of aggression in the future.

Another limitation is that in the present research, we only measured participants’ levels of forgiveness and did not manipulate it experimentally. In the future, it will be necessary to explore the role of forgiveness among ostracized people, for example, by using forgiveness intervention programs.

Furthermore, although we found that ostracism increases automatic aggression and the roles of anger and forgiveness in this relation, anger only partially mediated the effect (Study 3). It would be worthwhile in the future to investigate whether other factors have mediating or moderating effects, such as hostility, resilience, and hardiness. While some individuals are more likely than others to respond to ostracism with aggression, there are significant individual differences in aggressive responses (Ren et al., 2018). For example, hardiness is considered a pathway to resilience under stress: people facing challenges believe that stress is normal, whereas people with high control believe in trying to influence outcomes by the decisions they make (Maddi et al., 2011). As a result, we may reasonably predict that dispositional hardiness would moderate reaction to ostracism with aggression.

## Concluding Remarks

In the present research, we examined how ostracism influences automatic aggression. Specifically, we test whether ostracism increases automatic aggression and whether anger can account for this effect. Our research showed that ostracism leads individuals to associate the self with aggression and that this link is accounted for by anger; moreover, the effect of anger depends on an ostracized individual’s level of forgiveness. In contrast with controlled aggression, automatic aggression can be assessed by an implicit measure, which is less affected by social desirability and has discriminant validity in predicting aggression behavior. Future research should focus more on automatic aggression in examining the effect of ostracism on aggression.

## Data Availability Statement

The datasets generated for this study are available on request to the corresponding author.

## Ethics Statement

All studies were carried out in accordance with the recommendations of the Research Ethics Committee of Renmin University of China. All subjects in Studies 2 and 3 and all guardians of participants in Study 1 were given a written informed consent form in accordance with the Declaration of Helsinki and signed it.

## Author Contributions

DZ, SL, LS, and FT contributed to the conception and design. DZ, SL, LS, and AH contributed to the collection, analysis, and interpretation of data DZ and SL contributed to drafting the article. DZ, AH, FT, and KW contributed to revising the article critically.

### Conflict of Interest

The authors declare that the research was conducted in the absence of any commercial or financial relationships that could be construed as a potential conflict of interest.
